# University Teachers’ Decisions on Post-retirement Employment: Do Demographic Variables Count?

**DOI:** 10.1177/23337214211041419

**Published:** 2021-09-26

**Authors:** Inusah Salifu, Ebenezer D. Odame, Jibreel U. Abubakar

**Affiliations:** 1 Department of Adult Education and Human Resource Studies, School of Continuing and Distance Education, College of Education, 460920 University of Ghana, Accra, Ghana; 2 Department of Distance Education, School of Continuing and Distance Education, College of Education, University of Ghana, Accra, Ghana; 3 Department of Business Administration, University for Professional Studies, Accra, Ghana

**Keywords:** dependency, gender, Ghana, marital status, post-retirement decisions, university teachers

## Abstract

This research aimed to determine whether demographic characteristics of retiring university teachers were significantly associated with their post-retirement employment decisions, using three psychosocial theories–role theory, continuity theory and life course theory. The research used the cross-sectional survey design and relied on the simple random sampling technique to sample 235 participants who were left with 5 years or less to reach Ghana’s compulsory retirement age of 60 years. The participants were selected across 20 public universities in Ghana to complete a questionnaire. Based on a binary logistic regression analysis, the research found that, apart from the rank and health status of the participants, other variables such as gender, kind of university, family size, marital status and levels of involvement in university activities significantly determined the participants’ decisions regarding a working life after retirement. Against this backdrop, the research concluded that within the academic milieu, certain demographic factors were key determinants of post-retirement employment decisions.

## Introduction

This research was part of a large-scale investigation conducted to find out how retiring university teachers (i.e. those about to retire within 5 years) in Ghana intended to spend time after retirement. It aimed to determine whether key demographic variables such as gender, marital status, dependency, kinds of university, rank, level of involvement in university activities and health status were significantly associated with retiring university teachers’ post-retirement employment decisions (i.e. to either continue a working life with the same institutions or leave for other paid employments or non-paid occupations when they retire), using three psychosocial theories, namely, role theory (RT), continuity theory (CT) and life course theory (LCT). As applied to this research, occupation refers to any activity a person engages in regularly for either a gainful or non-gainful purpose. Although many literature sources (e.g. Smith & Moen, 2004; Statistics New Zealand, 2006; Stonebraker & Stone, 2015) have suggested that the concept of post-retirement is broad and multifaceted making it defy a precise definition, in this research, it is contextualised as the aftermath of compulsory retirement.

Globally, different countries have different retiring age limits for certain categories of public sector workers including teachers (Renner, 1987; Uccello, 1998). For example, the retiring age for all public sector workers in South Africa is 63 years (Marumoagae, 2017), but other African countries such as Ghana and Nigeria have their compulsory retirement age rather fixed at 60 years (Kusi et al., 2014). Outside the continent of Africa, the UK, for instance, has its retiring age for teachers in public schools at 65 years (Matthijs Bal & Visser, 2011) while in the Republic of Ireland, recent developments show that the country has reviewed its compulsory retiring age from 65 to 70 years for public sector workers (Given & Lawn, 2019).

In Ghana, the 60 years compulsory retiring age is a constitutional provision found in Clause 1, Article 199 of the 1992 Republican constitution. According to the provision, ‘A public officer shall, except as otherwise provided in this constitution, retire from the public service on attaining the age of 60 years’ (Constitution of the Republic of Ghana, 1992, p. 120). Unlike their counterparts in independent/privately owned universities, teachers in public universities in Ghana are classified as public workers and are, as a result, affected by the compulsory retiring age. However, those among them who are up to the rank of Senior Lecturer and above, and are in sound health, the law gives them a concession to do five more years on a voluntary post-retirement contract (Oteng et al., 2018). Until 2018, the five-year post-retirement limit was relaxed for university teachers who retired on the Professorial rank and those teachers could seek contract extension up to 10 years. Effective from 2018, however, the government applied the law strictly to make contract extension beyond the mandated five-year period for all retirees no more permissible unless a local unit of a university interested in hiring the person’s services engages the person as a consultant and pays him/her from funds generated internally.

It was necessary to conduct this research given that some aspects of the literature have documented that the experiences of people while in active work influence their desire for an earlier retirement and, subsequently, the kind of post-retirement employment decisions they would want to make. For example, workers who are happy in their employments are more likely to despise retirement and would not wish for it; and when they retire compulsorily, they are likely to accept post-retirement contracts with their current institutions (Fasbender et al., 2016; Hatcher, 2003; Naudé et al., 2009). On the contrary, workers who are less motivated in their present employments are likely to wish for an earlier retirement and await it with eagerness; and when eventually the time comes, they are less likely to decide on work continuity with current institutions (Solem et al., 2016; Urošević & Milijić, 2012).

Again, a good number of earlier studies such as Alafeef et al. (2011), Bashar et al. (2013), Kukanja (2013), Punj (2011), Urošević and Milijić (2012), Vallabh and Mhlanga (2014) and Zukowski and Brown (2007) have also posited that demographic variables of employees affect their performances while in active work, but it is not clear whether or not the variables also influence the kind of post-retirement employment decisions they would want to make.

### Theoretical Frameworks Underlying Work and Retirement Decisions

Cohen-Mansfield and Regev (2018) have explained three closely related psychosocial theories about work and retirement which are the following: role theory (RT) (Ashforth, 2000), continuity theory (CT) (Atchley, 1989, 1999) and life course theory (LCT) (Elder, 1998; Elder & Johnson, 2003; Giele & Elder, 1998). The RT is interested in how a role exit and a role transition influence the extent to which workers identify themselves with various roles for retirement outcomes (Cohen-Mansfield & Regev, 2018). By implication, workers who are very passionate about their roles and are very attached to them would naturally see transitions in the roles towards retirement as risky and dreaded phenomena.

The CT (Atchley, 1989, 1999) also argues that as human beings, workers would like to continuously enjoy financial status, skills, social relationships and functioning throughout lifetime (Cohen-Mansfield & Regev, 2018). As a result, their preference for a retirement transition is conditional on whether or not an opportunity exists for them to continue with already established life patterns (Beehr & Bowling, 2013; Bonsdorff & Ilmarinen, 2013; Cohen-Mansfield & Regev, 2018).

The LCT also known as the life course perspective theory or the life course approach have been explained extensively by Elder (1998), Elder and Johnson (2003) and Giele and Elder (1998). According to Giele and Elder, the theory refers to ‘a sequence of socially defined events and roles that the individual enacts over time’ (p. 22). Put differently, it examines how structural contexts as well as cultural and social change affect human beings generally over time but with a specific focus on those experiencing a working life. In particular, the theory conceptualises life course as age-differentiated social occurrences which are analysed based on a historical approach–an approach that explores how previous happenings shape and direct future decisions regarding social events such as employment, crime involvement, marriage or divorce (Wink & James, 2013).

From the above explications, it is apparent that all the three theories have asserted retirement is a psychosocial event and being so implies that decisions would have to be taken regarding how to spend the event. This assumption provides the basis for their application in this research on the influence of demographic factors on post-retirement employment decisions of retiring university teachers. Specifically, the RT is apt because of its emphasis on the influence of role exit and role transition on retirement decisions. In this current research, it will be used to analyse how the transition from various work roles such as Assistant Lecturer, Lecturer, Senior Lecturer and so on relates to and/or predicts the teachers’ post-retirement employment decisions. The CT is also appropriate given that it frames the teachers as human beings whose natural inclination should tilt towards maintenance of consistency in earlier patterns of employments. Finally, the LCT’s suitability stems from Cohen-Mansfield and Regev’s (2018) argument that the theory ‘…links individual attributes (e.g., demographics, health, coping skills, and abilities), social factors (such as age-based norms), roles, and individual history to individual adjustment’ (p. 428).

Consistent with the three theories, this research hypothesised that (H_1_) the demographic variables of employees such as retiring university teachers in Ghana would be significantly positively associated with their post-retirement employment decisions. This research had two objectives: The first was to examine the relationship between demographics of the teachers and their post-retirement employment decisions. The second was to predict the effect of the demographics on the decisions.

### Conceptual Framework

**Figure 1.**
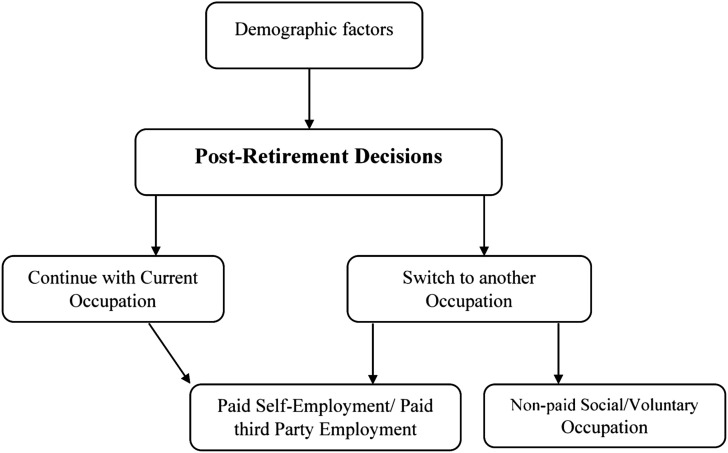
Post-retirement decisions (source: authors’ own creation).

The framework in Figure 1 depicts our conceptual understanding of how demographic variables are associated with post-retirement employment decisions. From the framework, it is assumed that demographic variables such as gender, marital status, dependency, faculty of affiliation, rank, level of involvement in university activities and health status may determine the decisions of employees to either continue a working life with their current institutions or leave for other paid and non-paid occupations when they retire.

## Methods

### Design and Participants

This research was conducted using the quantitative cross-sectional survey design to elicit the objective views of a large number of participants on post-retirement employment decisions (Creswell, 2009). The target population for the research was about 1124 retiring university teachers at the time of the survey in February 2021. Sampling was done in two stages. In the first stage, public universities in Ghana (*N* = 20) were purposively selected and categorised according to their predominant disciplines such as science, applied science and humanities. The institutions were chosen because they had the key informants required for the research. Independent/privately owned universities in the country were excluded from the research because they were not affected by the country’s constitutional provision mandating compulsory retirement at age 60 (see paragraph 3 of the introductory section).

In the second stage, the simple random technique was also used to sample 305 participants out of 895 accessible population (i.e. retiring university teachers who consented to taking part in the research). The sample size was considered representative because it met standard criteria for determining sample size (Krejcie & Morgan, 1970; Yamane, 1967). Although as per the criteria a total of 267 sample would have sufficed for the research, we opted to sample 305 participants instead to make up for missing responses and non-responses, given the nature of the data collection (see section on ‘Procedure’). Table 1 presents the socio-demographic characteristics of the participants.

**Table 1.** Demographic Features of Respondents.

Variables	No	%
Gender		
Male	114	48.5
Female	121	51.5
Total	235	100.0
Marital status		
Single	14	6.0
Married	179	76.2
Widowed	19	8.1
Divorced	18	7.7
Separated	5	2.1
Total	235	100.0
Kinds of university		
Predominantly science	144	61.3
Predominantly applied science	48	20.4
Predominantly humanities	43	18.3
Total	235	100.0
Rank		
Lecturer	119	50.6
Senior Lecturer	89	37.9
Assoc. Prof	9	3.8
Professor	18	7.7
Total	235	100.0
Level of involvement in university activities		
Highly involved	170	72.3
Somewhat involved	65	27.7
Total	235	100.0
Do you have any health challenges?		
No	215	91.5
Yes	20	8.5
Total	235	100.0

### The Instrument

The research used a self-designed instrument named ‘Post-Retirement Employment Decisions Questionnaire’ (PREDQ). The questionnaire was divided into two as follows: The first part had three items seeking information on relevant demographic data of respondents. The second part had five major items put in five sections (A, B and C) on the following themes, respectively: work continuity, alternative post-retirement employment activity and post-retirement non-employment activity. All items on the questionnaire were developed in accordance with authoritative views in the reviewed literature and “yes/no” and scale-type questions (Strongly Agree-5; Agree-4; Unsure-3; Disagree-2; Strongly Disagree-1) were mainly asked.

To ensure the validity of the instrument, a face validity test was conducted to find the effectiveness of the items in meeting the objective of the research (Johnson & Christensen, 2008), and the result was positive. A pilot test of the instrument was also done using 102 participants, and the result yielded a reliability co-efficient of 0.74 making the instrument reliable for use.

### Procedure

The data collection exercise took one and a half months to complete. The data collection process began with a formal request to the Human Resource (HR) offices of the selected universities for data on teachers about to retire within 5 years. After obtaining the needed information, a bulk email was sent to approximately 1124 target population explaining the nature of the research and inviting them to be participants. This was done albeit the HR offices had already briefed them about the survey and sought their consent before releasing their data to be included for sampling in this research. Eight hundred and ninety-five of the target population responded to the email, but the questionnaire was emailed to the sampled 305 teachers to complete. A total of 235 of the sample completed and returned the instrument and that gave a response rate of about 77%.

The data were analysed using SPSS version 25.0. Simple frequencies were generated, and cross tabulations and Pearson’s chi-square test of association were used to determine the relationship between variables while binary logistic regression analysis was computed to estimate the effect of socio-demographic variables on post-retirement intentions. To meet ethical standards, ethical clearance was obtained with approval number ECH 037/20-21 from one of the universities’ ethics committee for the humanities. Consent was sought and obtained from the participants before the research began. The identity of the participants was concealed, and their participation in the research was voluntary.

## Results and Discussion

This research hypothesised that (H_1_) the demographic variables of retiring university teachers in Ghana would be significantly positively associated with their post-retirement employment decisions. To test this hypothesis, a chi-square test was initially used to examine the relationship between demographics of the teachers and their post-retirement employment decisions. To further predict the effect of the former on the latter, logistics regression (LR) was also employed.

### Relationship Between Demographics and Decisions

Table 2 presents a cross-tabulation and a chi-square test conducted to assess the relationship between socio-demographic characteristics of the retiring university teachers and their decisions to continue a working life with original institutions or leave for other paid employments or non-paid occupations. From the table, respondents’ gender significantly relates to their decision to continue a working life with current institutions (*P* < .05). For instance, more females (52.1%) than males (38.6%) resolved that they would seek or accept a post-retirement contract with their current institutions. Additionally, marital status (*P* < .05), family size (*P* < .05) and respondents’ level of involvement in university activities (*P* < .05) all show a significant relationship with the decision. There are, however, relativities. For example, the majority of respondents who were divorced (77.8%) indicated the decision compared with those who were widowed (21.1%), married (46.9%) or single (35.7%). Again, the majority of respondents with a family size of four or more (59.1%) similarly took the decision compared with those with family sizes of less than 4 (32.5%). These findings buttress and reinforce the assertion that demographic factors of employees have a significant influence on their attitudes and behaviour at work (Bashar et al., 2013; Kukanja, 2013; Urošević & Milijić, 2012). However, the finding that more females than males decided on work continuity with their original institutions when they retired is inconsistent with Pleau and Shauman’s (2013) assertion that women are less likely to re-enter the labour force after retirement. From continuity (Atchley, 1989, 1999) and life course (Elder, 1998; Elder & Johnson, 2003; Giele & Elder, 1998) theoretical perspectives, these results depict the normal passage of human life with time and the quest to maintain a pattern of life. The theories argue that human beings naturally prefer stability of interpersonal relationship, occupation and financial status throughout a lifetime (Bonsdorff & Ilmarinen, 2013). The life course theory, in particular, posits that human attributes such as demographics are inextricably connected to the decision people make about social life.

**Table 2.** Socio-Demographic Characteristics by Decision to Seek/Accept a Post-Retirement Contract.

Socio-demographic Variables	Decision to seek/accept a contract with current University		Total
	Will not seek/accept a contract	Will seek/accept a contract	*N* = 235, (100%)
	*n* = 128, % = (54.5%)	*n* = 107, % = (45.5%)	
Gender			
Male	70 (61.4%)	44 (38.6%)	114 (100.0%)
Female	58 (47.9%)	63 (52.1%)	121 (100.0%)
χ^2^ statistics = 4.29, df (1), P = 0.04 < .05			
Marital			
Single	9 (64.3%)	5 (35.7%)	14 (100.0%)
Married	95 (53.1%)	84 (46.9%)	179 (100.0%)
Widowed	15 (78.9%)	4 (21.1%)	19 (100.0%)
Divorced	4 (22.2%)	14 (77.8%)	18 (100.0%)
Separated	5 (100.0%)	0 (0.0%)	5 (100.0%)
χ^2^ statistics = 17.00, df (4), P = .002 < .05			
Kinds of university			
Predominantly science	81 (56.2%)	63 (43.8%)	144 (100.0%)
Predominantly applied science	28 (58.3%)	20 (41.7%)	48 (100.0%)
Predominantly humanities	19 (44.2%)	24 (55.8%)	43 (100.0%)
χ^2^ statistics = 2.31, df (2), P = .32 > .05			
Rank			
Lecturer	64 (53.8%)	55 (46.2%)	119 (100.0%)
Senior Lecturer	59 (66.3%)	30 (33.7%)	89 (100.0%)
Associate Professor	5 (55.6%)	4 (44.4%)	9 (100.0%)
Professor	0	18 (100.0%)	18 (100.0%)
χ^2^ statistics = 0.25, df (1), P = .62 > .05			
Level of involvement in university activities			
Highly involved	103 (60.6%)	67 (39.4%)	170 (100.0%)
Somewhat involved	25 (38.5%)	40 (61.5%)	65 (100.0%)
χ^2^ statistics = 9.28, df (1), P = .002 < .05			
Health challenges			
No health challenges	123 (57.2%)	92 (42.8%)	215 (100%)
Have health challenges	5 (25.0%)	15 (75.0%)	20 (100%)
χ^2^ statistics = 7.65, df (1), P = .006 < .05			
Family size			
Less than 4	81 (67.5%)	39 (32.5%)	120 (100.0%)
4 or more	47 (40.9%)	68 (59.1%)	115 (100.0%)
χ^2^ statistics = 16.79, df (1), P = .0001 < .05			

Further, although the results show that the rank of respondents had no significant relationship with their decisions to continue a working life with their current institutions, a comparison indicates that all the respondents who were professors decided that they would seek or accept a post-retirement contract with their current universities. This revelation perhaps reaffirms the common saying that professors ‘never retire’ literally meaning that professors, unless incapacitated, continue their mentoring roles even after their retirement and academia loses their knowledge and experiences if they do not accept post-retirement work contracts (Beehr et al., 2000; Dorfman et al., 1984; Renner, 1987; Stonebraker & Stone, 2015). It also supports Ashforth’s (2000) role theory which mainly assumes that employees who are very desirous of their roles would naturally want to maintain them throughout their lifetimes.

On the health status of respondents, the results again point to a significant relationship with the decision to continue a working life with current employers (*P* < .05). For instance, a higher percentage of respondents who stated that they had health challenges (75%), compared to 42.8% of respondents who had no health challenges, indicated that they would seek or accept a post-retirement contract with their present universities. By implication, this part of our results means that the majority of respondents had resolved to continue an active working life even when they retire regardless of their health challenges. We find this outcome not only novel but also curious and intriguing because the decision to work after retirement, whether with the current institution or switch to another despite health challenges, is normally not expected of workers with health issues. On the contrary, one would anticipate such people to rather prefer early retirement, and even if they stay on until actual retirement time, consider the occasion as a rare opportunity to rest and/or seek medical attention to recuperate. This assumption is premised on the fact that most aged people have increasing health needs. Based on this finding, we may only speculate that the situation might have arisen because of the inability of most university teachers in Ghana to mobilise enough savings towards retirement. We further argue that in Ghana, health challenges do not necessarily pose a serious barrier to university teachers who are desirous of continuing a working life when they retire.

Additionally, the kind of university where the teachers taught (*P* > .05) did not significantly relate to their post-retirement employment decisions albeit there were slight differences in the percentage of those who were willing to do so. For example, a higher percentage of respondents from predominantly humanities universities (55.8%) than from predominantly science universities (43.8%) and predominantly applied science universities (41.7%) had decided on seeking a post-retirement contract with their current universities. This aspect of the results somewhat negates the assumptions of the three theories underpinning this research, particularly the life course theory which emphasises the influence of social structures on choices of life pattern (Cohen-Mansfield & Regev, 2018). Despite this, the result may be expected given that, in Ghana, all public universities have very similar conditions of service.

### Predicting the Effect of Demographics on Decisions

The results in Table 3 show the outcome of a binary logistic regression estimating the effect of socio-demographic variables such as gender, marital status, kind of university, rank, level of involvement in university activities and family size of the retiring university teachers on their post-retirement employment decisions. The Nagelkerke *R*^2^ value of 0.45 suggests that the model correctly predicts 45% of the variance in the outcome variable, and the Hosmer and Lemeshow test which is significant at a 5% level of significance shows that these sets of selected predictor variables better predict the likelihood of respondents’ decisions to seek or accept a contract with their current universities to continue teaching and research when they retire.

**Table 3.** Summary of Results on Binary Logistic Regression Coefficients.

Predictors	B	S.E.	Wald	Exp(β)	95% C.I. for Exp(β)	
					Lower	Upper
Gender						
Male (RC)	—	—	—	1.00	—	—
Female	1.521	.377**	16.247	4.576	2.184	9.588
Kind of university						
Science universities	−1.061	.487*	4.746	.346	.133	.899
Applied science universities	**−**.642	.548	1.371	.526	.180	1.541
Humanities universities (RC)	—	—	—	1.00	—	—
Rank						
Lecturer (RC)	—	—	—	1.00	—	—
Senior Lecturer	**−**.042	.413	.010	.959	.426	2.156
Associate Professor	1.325	.848	2.438	3.761	.713	19.833
Professor	21.563	9050.678	.000	2,314,638.2	0.000	—
Level of involvement in university activities						
Highly involved	1.936	.483**	16.030	6.928	2.686	17.868
Somewhat involved (RC)	—	—	—	1.00	—	—
Family size						
Less than 4	—	—	—	1.000	—	—
4 or more (RC)	1.338	.389**	11.813	3.811	1.777	8.174
Marital status						
Single (RC)	—	—	—	1.00	—	—
Married	−.793	.745	1.131	.453	.105	1.950
Widowed	−2.695	1.060**	6.467	.068	.008	.539
Divorced	.259	.950	.075	1.296	.201	8.340
Separated	−21.90	17,974.843	.000	.000	0.000	—
Health challenges						
No health challenges (RC)	—	—	—	1.00	—	—
Have health challenges	−.232	.750	.096	.793	.182	3.45

*Note.* R2 = 0.45 (Nagelkerke; Cox & Snell) = 0.34, χ = 28.02, df (7), *P* < .05 (Hosmer & Lemeshow), goodness-of-fit χ2 (8) = 17.93, *P* = .02; −2 log likelihood = 227.214, χ2 (12) = 96.69, *P* = .0001 (Omnibus test of model coefficient), **P < .01, *P < .05.

In addition, the Omnibus test of model coefficient computed rejects the hypothesis that suggests that the model is not a significant fit of the data (χ^2^ = 96.686, *P* = .0001 < .05). Also, the overall predictive accuracy of the model is 80.0%. In terms of the predictor variables selected, gender (*P* < .05), kinds of university (*P* < .05), level of respondents’ involvement in university activities (*P* < .05), family size (*P* < .05) and marital status (*P* < .05) significantly predict the outcome variable of the model. Given this, the study accepts the hypothesis which states that socio-demographic variables such as gender, institution, family size, marital status and respondents’ level of involvement in university activities have significant effects on post-retirement employment decisions. Conversely, the study rejects the hypothesis stating that the rank (*P* > .05) and health status (*P* > .05) of respondents have no significant effect on the decisions, given that the two variables did not significantly predict the outcome of the model.

Furthermore, the results show that the likelihood of seeking or accepting a contract increases among females than males (β = 1.521, *P* < .05). For instance, females were about five times more likely to seek or accept a contract with their universities when they retire than their male counterparts (OR = 4.576, CI = 2.18–9.59). Given that in the African context, male spouses are husbands who take up financial responsibility for family upkeep and female spouses often play a supporting role as wives even amid the current trend of changing gender roles, this result is baffling because one would have expected that more males than females would rather have opted to seek or accept a post-retirement contract with current institutions because of their financial burden. However, in the context of this study, financial motivation was not stated as the reason why respondents would seek or accept a post-retirement contract with their current employers. In contemporary times, many African women, to a large extent, are being motivated to pursue their career to the fullest in order to also serve as role models to other younger females who would want to pursue a career in the academia. Consequently, female retiring university teachers would have a higher tendency to seek or accept a post-retirement contract with their employers to continue mentoring other females in their institutions. In terms of institutional dynamics, the results again show that the likelihood of seeking or accepting a post-retirement contract with original institutions decreased among teachers from predominantly science universities than teachers from predominantly humanities universities (β = −1.061, *P* < .05). Further details indicate that, teachers from predominantly science universities were three times less likely to seek or accept a post-retirement contract than those from predominantly humanities universities (OR = 1/0.346, CI = 0.13–0.90). This result may serve to explain the fact that due to the practical nature of teaching science, it requires teachers to spend a lot of time conducting laboratory experiments and engaging in fieldworks with students. The stress of experiments and fieldworks could influence the teachers to avoid post-retirement contracts to work in the same role. While on retirement, they may rather prefer to work in less tiring and demanding jobs whose returns could also serve as a buffer against some of the harsh socio-economic conditions associated with old age, limited income sources and access to quality health care in developing countries, especially Africa (Cohen-Mansfield & Regev, 2018; Zukowski & Brown, 2007).

Unsurprisingly, the results further show that the likelihood of seeking or accepting a post-retirement contract rather increased among teachers who were highly involved in university activities relative to those who were somewhat involved (β = 1.936, *P* < .05). To be precise, the results reveal that such active teachers were seven times more likely to seek or accept a post-retirement contract than those who were not highly involved (OR = 6.93, CI = 2.69–17.87). This may be due to the stronger interest they have in the institutions they belong. Research has also shown that teachers who are highly involved in institutional activities, whether for teaching, research or management purposes, stand a better chance of being considered for offers in their institutions (Hatcher, 2003; Naudé et al., 2009; Solem et al., 2016) perhaps, including a post-retirement contract. The favour becomes even more obvious when such active teachers retire on senior managerial positions.

Regarding family size or dependents, the results show that teachers with four or more dependents were four times more likely to seek or accept a post-retirement contract than those with a family size of less than four (OR = 3.81, CI = 1.78–8.17). Given the financial burden associated with catering for large family sizes, it is not surprising that university teachers with four or more dependents were more likely to avail themselves of post-retirement employments. Although this research, to some extent, does not solely associate financial motive with the desire among university teachers in Ghana to seek post-retirement employments, in circumstances where a determinant such as family size affects post-retirement employment decisions, the study suggests that it could be financially driven. Thus, post-retirement employments do not only help to cater for both economic and social safety nets of the teachers, but they also assist to meet the goals of their respective institutions.

Lastly, in terms of marital status, the results in Table 3 show that the likelihood of seeking or accepting a post-retirement contract decreased among faculty who were widowed than those who were single (β = −2.695, *P* < .05). For example, respondents who were widowed were 15 times less likely to accept/seek a post-retirement contract compared with respondents who indicated that they were single (OR = 1/0.068, CI = 0.008–0.539).

## Conclusion and Recommendation

This research examined the relationship between demographics of retiring university teachers in Ghana and their post-retirement employment decisions. It also predicted the effect of the demographics on the decisions based on assumptions of the theories. The preliminary finding revealed that all the demographic variables, except rank and the kind of university where a retiring university teacher taught, significantly related to the retiring university teachers’ post-retirement employment decisions. Consistent with the theories, the research further found that all the variables, except rank and health status variables, predicted the teachers’ post-retirement employment decisions. Quite notably, of all the variables, it was only the rank of the university teachers that did not significantly relate to and predict the teachers’ post-retirement employment decisions. The major limitation of this research is its design as a cross-sectional survey which does not allow an opportunity for causal interpretations. Nonetheless, the findings are unique given that, to the best of our knowledge, no previous research has established whether there is a connection between demographic variables of employees and the decisions they make regarding a working life after retirement. It also contributes to the burgeoning literature on human resource management in higher education because it proffers preliminary results suggesting that indeed, except for university teachers’ rank and health status, all other demographic variables such as gender, institution, family size, marital status and respondents’ levels of involvement in university activities may have a far-reaching influence on the decisions they make regarding a working life after retirement.

Further, although this research upholds the common belief that retirement is a welcoming event in the life of many people engaged in active labour, it argues that certain demographic factors significantly affect this event. The research has also highlighted the fact that university teachers who are highly involved in the activities of their institutions are most likely to accept or seek a post-retirement contract with institutions. Again, the research has brought to light that although both work and non-work-related factors contribute to post-retirement decisions of university teachers, the influence of demographic variables on gaining a better appreciation of why these decisions are made cannot be overlooked.

Overall, this research was conducted using only the quantitative approach. To shed light on the quantitative data, a future research may use the mixed-method approach because triangulating these methods would provide a deeper understanding of the post-retirement employment decisions of university teachers. For instance, why female university teachers have a higher likelihood of seeking or accepting a post-retirement contract is an area worth exploring further using qualitative approaches. The gender implication and the intersection of gender with other key variables such as age, marital status, dependency, rank and kind of university, health status and level of involvement would be an interesting area for further studies.

To compare contextual experiences of university teachers, further research involving university teachers from multiple countries with a similar working condition may also be a necessary future effort. The research examined only the influence of demographic variables on post-retirement decisions of employees. It is recommended that further research be conducted to examine the influence of other independent variables such as financial status, organisational culture, job characteristics, personality and belief systems on the post-retirement decision of universities.

## Supplemental Material

sj-pdf-1-ggm-10.1177_23337214211041419 – Supplemental Material for University Teachers’ Decisions on Post-retirement Employment: Do Demographic Variables Count? Click here for additional data file. Supplemental Material, sj-pdf-1-ggm-10.1177_23337214211041419 for University Teachers’ Decisions on Post-retirement Employment: Do Demographic Variables Count? by Inusah Salifu, Ebenezer D. Odame and Jibreel U. Abubakar in Gerontology and Geriatric Medicine
