# Supplementary material for: University Teachers’ Decisions on Post-retirement Employment: Do Demographic Variables Count?
Source: Gerontol Geriatr Med. 2021 Sep 26;7:23337214211041419. doi: 10.1177/23337214211041419 (PMC8477707; doi:10.1177/23337214211041419)
Supplement: sj-pdf-1-ggm-10.1177_23337214211041419 – Supplemental Material for University Teachers’ Decisions on Post-retirement Employment: Do Demographic Variables Count? [file sj-pdf-1-ggm-10.1177_23337214211041419.pdf]

UNIVERSITY TEACHERS' DECISIONS ON POST-RETIREMENT EMPLOYMENT: DO DEMOGRAPHIC VARIABLES COUNT?

This questionnaire aims to collect research information from lecturers retiring within the next five years on decisions regarding how they intend to spend an employment or a non-employment life after retirement. The research is being conducted solely for academic purposes, and the information that you provide will be treated with utmost confidentiality. Thanks for your participation.

PART ONE: DEMOGRAPHIC BACKGROUND

Please indicate your choice with a tick (✓)

1. **Gender:** Male ( ); Female ( )
2. **Marital status**: Married ( ); Single ( ); Divorced ( ); Separated ( ); Widowed( )
3. **Number of family dependents**: 1 ( ); 2 ( ); 3 ( ); 4 ( ); 5( ); above 5( )
4. **University:**
5. **Rank:**
6. **Level of involvement in University activities: Highly involved ( ); Somewhat involved ( ); Not involved ( )**
7. **Do you have any health challenges: Yes ( ); No ( )**

PART TWO: POST-RETIREMENT DECISIONS

SECTION A: WORK CONTINUITY

8. Do you intend to seek/accept a contract with **<u>your current university</u>** to continue teaching and research when you retire? (Please indicate your choice with a tick (√)

Yes ( )

No ( )

If your answer to question 9 is "Yes", please give reasons for your response and end here

………………………………………………………………………………………
………………………………………………………………………………………
………………………………………………………………………………………
………………………………………………………………………………………
………………………………………………………………………………………
………………………………………………………………………………………

If your response to question 9 is “No”, please proceed to Sections B and C and end there but skip to Sections D and E if you do not intend to at all be employed when you retire.

SECTION B: ALTERNATIVE POST-RETIREMENT EMPLOYMENT ACTIVITY

9. Instead of working with my current university when I retire, I rather intend to… (Please tick (√)

**Variable**	**Strongly disagree**	**Disagree**	**Unsure**	**Agree**	**Strongly agree**
Teach in another institution for another employer					
Do a personal research					
Do research for another employer					
Engage in consultancy					
Invest in securities (e.g., shares, gold, treasury bills)					
Do trading					
Establish a school					
Establish a medical/health centre/pharmacy					
Operate a transport business					
Write books					
Engage in estate business					
Do farming					
Engage in political party activism					

Please give other post retirement activities you will engage yourself in not specified on the table

…………………………………………………………………………………………………

………………………………………………………...

....………………………………

…………………………………………………………………………………………………….

SECTION C: POST-RETIREMENT NON-EMPLOYMENTACTIVITY

10. Instead of an employment life, I rather intend to… (Please tick (√))

**Variable**	**Strongly disagree**	**Disagree**	**Unsure**	**Agree**	**Strongly agree**
Travel around to pass time					
Spend time with relatives and acquaintances					
Sports					
Engage in philanthropy (e.g., running a charity organisation)					
Engage in religious devotions (e.g., preaching, teaching the gospel					
Do other volunteer services (e.g., counselling and/or mentoring younger people)					
Be in advocacy (e.g., championing minority					

rights, rights of vulnerable people)					
Engage in music production					
Engage in political party activism					
Engage in a fitness club					

Please give other post retirement activities you will engage yourself in not specified on the table

……

…………………………………………………………………...

....………………………………

…….
